# Willingness to vaccinate against COVID-19: the role of health locus of control and conspiracy theories

**DOI:** 10.1192/j.eurpsy.2022.1249

**Published:** 2022-09-01

**Authors:** V. Pisl, J. Vevera

**Affiliations:** 1 Faculty of Medicine and University Hospital in Pilsen, Charles University, Department Of Psychiatry, Plzen, Czech Republic; 2 Faculty of Medicine in Pilsen, Charles University, Department Of Psychiatry, Plzen, Czech Republic

**Keywords:** health locus of control, vaccination, Covid-19, conspiracy theories

## Abstract

**Introduction:**

Understanding the predictors of the willingness to get vaccinated against COVID-19 may aid in the resolution of current and future pandemics.Understanding the predictors of the willingness to get vaccinated against COVID-19 may aid in the resolution of current and future pandemics.

**Objectives:**

We aim to investigate how the readiness to believe conspiracy theories and the three dimensions of health locus of control affect the attitude towards vaccination.

**Methods:**

A cross-sectional study was conducted based on data from an online survey of a sample of Czech university students (n=866) collected in January 2021, using multivariate linear regression models and moderation analysis.

**Results:**

Sixty-six percent of Czech students wanted to get vaccinated against COVID-19. Forty percent of the variance of willingness to get vaccinated was explained by the belief in covid-related conspiracy theories and the powerful others dimension of health locus of control. One sixth of the variance of the willingness to get vaccinated was explained by health locus of control, cognitive reflection, and digital health literacy.

**Conclusions:**

Health locus of control and conspiracy mentality and its predictors are valid predictors of a hesitancy to get vaccinated against COVID-19. Campaigns promoting vaccination should target groups specifically vulnerable to conspiracy theories and lacking health locus of control related to powerful others.

**Disclosure:**

No significant relationships.

